# Many ways of communication: from *Helicobacter pylori *adherence to death, disruption, migration and escape

**DOI:** 10.1186/1478-811X-9-24

**Published:** 2011-11-01

**Authors:** Silja Wessler

**Affiliations:** 1 Division of Microbiology, Paris-Lodron University, Salzburg, Austria

## 

It is almost 30 years ago that *Helicobacter pylori *(*H. pylori*) was identified as an important causative agent in the development and progression of a wide range of gastro-intestinal diseases including acute and chronic gastritis, duodenal ulceration, MALT lymphoma and gastric cancer [1,2]. During this time much progress has been made in understanding the complex mechanisms of bacterial pathogenesis which is summarized in this special issue 'Interaction of *Helicobacter pylori *with its host cell'.

The outcome of gastric disorders is determined by the highly coordinated interaction of bacterial pathogenic factors with signal transduction pathways in host cells leading to diverse cellular responses [3]. Reflecting bacterial pathogenesis as a complex regulated multi-step process *H. pylori *induces disruption of intercellular adhesions, cell motility, apoptosis and the escape from the immune system [4]. The initial step is the bacterial adherence to epithelial host cells. Backert and colleagues [5] describe the molecular interaction of important adherence factors such as BabA/B, SabA, AlpA/B, OipA, and HopZ establishing a tight bacterial contact with host target cells necessary for colonization and pathogenesis of *H. pylori*. While receptors for BabA and SabA have already been identified, the respective binding partners on host cells for OipA, HopZ and AlpA/B are unknown. In this issue, Backert et al. further focus on the selective interaction of *cag*PAI (*cag *pathogenicity island) components with integrin β1 presented on the surface of gastric epithelial cells [5]. The *cag*PAI protein CagL decorates the tip of the type IV secretion system and directly binds to β1 integrin *via *an arginine-glycine-aspartate (RGD) motif. This triggers the delivery of the pathogenic factor CagA (cytotoxin associated gene A) into the cytoplasm of host cells as well as activation of β1 integrin signaling [6]. Both, integrin β1 signaling and CagA injection are crucially important in *H. pylori *pathogenesis as they induce a strong motility and invasive growth of infected epithelial cells [7,8]. As summarized in the review of Wessler et al. the well-studied interplay of signal transduction pathways initiated by CagL/integrin β1 binding and CagA is considered as a significant mediator of the dynamic rearrangements in the actin cytoskeleton in *H. pylori*-infected cells *in vitro*. These factors activate a complex network of signal transduction pathways involving different host cell kinases, adaptor proteins, GTPases, and actin binding proteins and leading to the deregulation of the actin cytoskeleton and actin-dependent processes such as the formation of lamellipodia, invadopodia and, finally, in combination with proliferative processes, invasive growth [7].

Another important aspect in the step-wise bacterial pathogenesis is the active disruption of lateral cell-to-cell contacts, which perturbs the integrity of the gastric barrier. In this context, tight junctions and adherence junctions are intensively studies and the current knowledge is presented by Wroblewski and Peek in this special issue [9]. Intact intercellular contacts are required to establish and maintain the molecular architecture and selective barrier function of a healthy polarized epithelial tissue. Through the direct interference with components of the junction complexes, *H. pylori *can actively disturb the barrier function. The bacterial factors VacA, OipA, urease, HtrA, and CagA appear to be involved in the loss of intercellular adhesions which affect tight junctions and adherence junctions extra- or intracellularly as discussed in the review by Wroblewski et al. [9].

Apoptosis is a frequently observed cell response to *H. pylori *after long-term infections which contribute to the disruption of the barrier function and subsequently to pathogenesis. Apoptosis has been closely related to another important virulence factor, the vacuolating cytotoxin A (VacA), of *H. pylori *[10,11]. Once secreted into the environment of *H. pylori*, it enters the eukaryotic host cells exhibiting pleiotropic functions such as vacuolation, suppression of the immune system and the induction of apoptosis. The review by Rassow describes the intracellular traffic route of VacA from the host cell membrane to mitochondria where VacA forms chloride channels, mediates loss of the mitochondrial membrane potential, recruits Bax and Bak, and induces the release of cytochrome c to promote apoptosis [12].

The chronic inflammation in *H. pylori*-infected individuals significantly enhances the risk of cancer development [13]. Although the immune system is stimulated, persistent infection occurs. This is made possible by the failure of the immune system to clear the *H. pylori *infection pointing to an immensely successful strategy of immune evasion. The review by Müller and colleagues summarizes the immune cell types and signaling pathways that are involved in establishing persistent infection. Pathogen-associated molecular patterns (PAMPs) and their pattern recognition receptors (PRR), the escape from the T cell-mediated adaptive immunity and reprogramming of the immune system are discussed. Another aspect considered in this review is the growing recognition of the role of *H. pylori *in the prevention of allergic and auto-immune diseases in chronically infected individuals [14].

The current knowledge of *H. pylori *pathogenesis is still incomplete, but already points to a strictly regulated multi-step process. This special issue provides a comprehensive overview of presently known *H. pylori*-activated signal transduction pathways leading to different cellular processes and emphasizes the complexity of the communication between *H. pylori *and its host.
